# An early novel prognostic model for predicting 80-day survival of patients with COVID-19

**DOI:** 10.3389/fcimb.2022.1010683

**Published:** 2022-10-27

**Authors:** Yaqiong Chen, Jiao Gong, Guowei He, Yusheng Jie, Jiahao Chen, Yuankai Wu, Shixiong Hu, Jixun Xu, Bo Hu

**Affiliations:** ^1^ Department of Laboratory Medicine, Third Affiliated Hospital of Sun Yat-sen University, Guangzhou, Guangdong, China; ^2^ Department of Infectious Diseases, Key Laboratory of Liver Disease of Guangdong Province, Third Affiliated Hospital of Sun Yat-sen University, Guangzhou, Guangdong, China; ^3^ Department of Laboratory Medicine, Huangshi Hospital of Traditional Chinese Medicine (TCM) (Infectious Disease Hospital), Huangshi, Hubei, China

**Keywords:** COVID-19, nomogram, prognosis, predict, survival

## Abstract

The outbreak of the novel coronavirus disease 2019 (COVID-19) has had an unprecedented impact worldwide, and it is of great significance to predict the prognosis of patients for guiding clinical management. This study aimed to construct a nomogram to predict the prognosis of COVID-19 patients. Clinical records and laboratory results were retrospectively reviewed for 331 patients with laboratory-confirmed COVID-19 from Huangshi Hospital of Traditional Chinese Medicine (TCM) (Infectious Disease Hospital) and Third Affiliated Hospital of Sun Yat-sen University. All COVID-19 patients were followed up for 80 days, and the primary outcome was defined as patient death. Cases were randomly divided into training (n=199) and validation (n=132) groups. Based on baseline data, we used statistically significant prognostic factors to construct a nomogram and assessed its performance. The patients were divided into Death (n=23) and Survival (n=308) groups. Analysis of clinical characteristics showed that these patients presented with fever (n=271, 81.9%), diarrhea (n=20, 6.0%) and had comorbidities (n=89, 26.9.0%). Multivariate Cox regression analysis showed that age, UREA and LDH were independent risk factors for predicting 80-day survival of COVID-19 patients. We constructed a qualitative nomogram with high C-indexes (0.933 and 0.894 in the training and validation groups, respectively). The calibration curve for 80-day survival showed optimal agreement between the predicted and actual outcomes. Decision curve analysis revealed the high clinical net benefit of the nomogram. Overall, our nomogram could effectively predict the 80-day survival of COVID-19 patients and hence assist in providing optimal treatment and decreasing mortality rates.

## Introduction

As of July 24, 2022, just under 567 million confirmed cases of COVID-19 and a global death toll exceeding 6.3 million deaths had been reported globally (https://www.who.int) (2022). Severe Acute Respiratory Syndrome coronavirus 2 (SARS-CoV-2) is the cause of the serious life-threatening disease known as COVID-19 (Liu et al., 2020a). COVID-19 mortality is intricately linked to the lack of access to specific therapeutic agents or vaccines (Cascella et al., 2020), given that the global health system is significantly burdened by this pandemic (Momtazmanesh et al., 2020).

Most patients have mild or common symptoms and can be discharged after symptomatic treatment. However, some patients may require further hospitalization with disease progression, presenting critical symptoms or complications, such as dyspnea, hypoxemia and acute respiratory distress syndrome (Huang et al., 2020; Zhu et al., 2020). Owing to the rapid spread of COVID-19, medical resources in many countries, especially the intensive care unit (ICU), are being over-requisitioned and almost exhausted (Wu et al., 2020b). Therefore, decreasing COVID-19-related deaths and alleviating the burden on overloaded medical facilities emphasize the need for a model for early prediction of severe disease progression and death. Many risk factors associated with severe COVID-19 disease progression have been identified, including comorbidity, older age, lower lymphocyte and higher lactate dehydrogenase (LDH), viral load and so on (Gong et al., 2020; Ji et al., 2020; Pujadas et al., 2020). Subsequently, these risk factors were harnessed to construct prediction models (Li et al., 2020a; Wu et al., 2020a; Zhou et al., 2020), such as CALL score and Patient Information Based Algorithm (PIBA) (Ji et al., 2020; Wang et al., 2020b; Schiaffino et al., 2021).

A nomogram is a graphical representation of predictive statistical models for individual patients and also an alternative method for various types of diseases (Gong et al., 2020; Li et al., 2020b; Liu et al., 2020b; Xu et al., 2020). However, few nomograms have hitherto been developed to predict the prognosis of COVID-19 patients. In this retrospective study, we analyzed the laboratory tests of COVID-19 patients and constructed a nomogram to predict the prognosis more accurately based on baseline data from two clinical centers. Importantly, our nomogram could guide clinicians in predicting the death risk of COVID-19 patients, providing early intervention, prioritizing medical resources and reducing mortality.

## Materials and methods

### Data collection

Clinical records and laboratory results were retrospectively reviewed for 331 patients with COVID-19 from Huangshi Hospital of Traditional Chinese Medicine (Infectious Disease Hospital) and Third Affiliated Hospital of Sun Yat-sen University between January 20, 2020 and May 20, 2020. Patients younger than 14 years of age were excluded from the study. Two patient between 14 to 15 years old was included in this study. Demographic data, including age, sex, clinical signs and symptoms such as fever and diarrhea, presence of comorbidities and clinical laboratory test results, were all collected upon admission. All included COVID-19 patients were followed up for 80 days on admission by phone to determine whether they survived (Survival group) or not (Death group).

The study was approved by the Ethics Committee of Third Affiliated Hospital of Sun Yat-sen University and the Ethics Commission of Huangshi Infectious Disease Hospital and exempted from informed consent given the retrospective nature of this study.

Patients diagnosed with COVID-19 were included in the study. The diagnosis of SARS-CoV-2 infection has been described previously (Gong et al., 2020). A confirmed case was defined as an individual with laboratory-confirmed SARS-CoV-2, which required positive results of SARS-CoV-2 RNA, regardless of clinical symptoms and signs.

### Laboratory methods

The clinical laboratory examination results of patients for white blood cell (WBC), red blood cell (RBC), hemoglobin (HGB), blood platelet (PLT), neutrophils, lymphocyte, neutrophil-lymphocyte ratio (NLR), monocyte, international normalized ratio (INR), albumin (ALB), C-reactive protein (CRP), direct bilirubin (DBIL), UREA, lactate dehydrogenase (LDH), glucose (GLU) were collected. All biochemical parameters were obtained through standard automated laboratory methods and commercial kits in accordance with the instrument operating procedures.

### Statistical analysis

Categorical variables were expressed as frequency and percentage; continuous variables as mean (standard deviation [SD]) or median (interquartile spacing [IQR]), as appropriate. The Fisher exact test was used to analyze the significance of Categorical variables. The Student’s t-test was used to compare continuous variables with a normal distribution. The Mann-Whitney U test was used for continuous variables with a non-parametric distribution. SPSS 22.0 statistical software package (SPSS, Inc., Chicago, IL, USA) was used for the above statistical analysis. To determine the relative importance of each feature, the Least Absolute Shrinkage and Selection Operator (LASSO) was used for feature selection, and the logistic regression was used to establish the regression prediction model.

To minimize bias of the regression coefficient, predictors with a missing rate of more than 5% were excluded. As previously described (Gong et al., 2020), the missing values were imputed by the expectation-maximization (EM) method using SPSS statistical software. The nomogram was established with the rms package, and the performance of the nomogram was evaluated by discrimination (Harrell’s concordance index) and calibration (calibration plots and Hosmer-Lemeshow calibration test) analyses in R. R software (version 3.6.2) was used for all statistical analysis except SPSS analysis. The optimal cut-off value of total points of the nomogram was determined by the R package survminer, which uses the maximally selected rank statistics from the ‘maxstat’ R package. Survival curves were depicted by the Kaplan-Meier analysis and compared by the log-rank test. A P-value < 0.05 was statistically significant.

## Results

### Demographics and characteristics of COVID-19 patients

**Figure 1:**
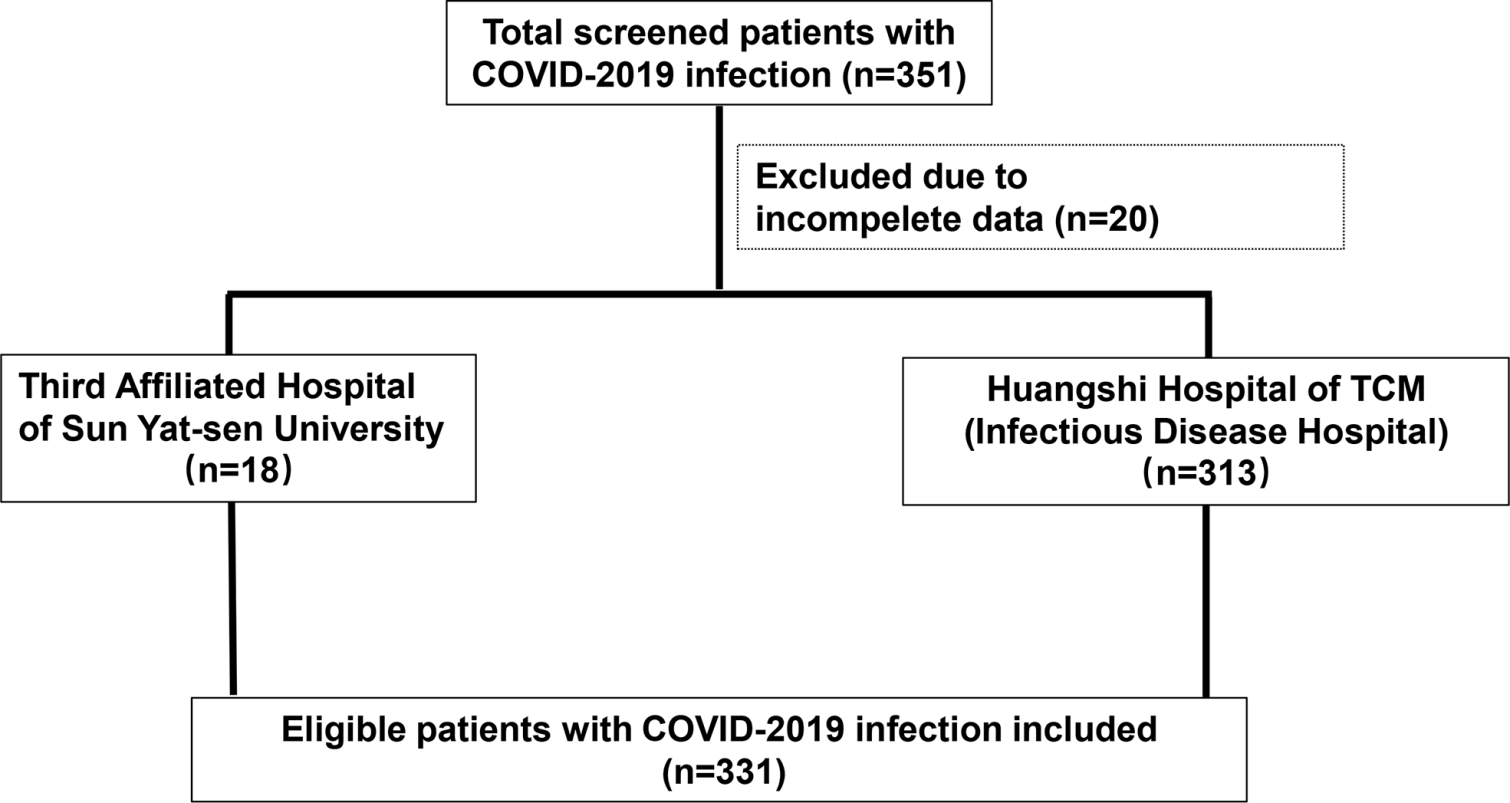
Flow chart of enrolled patients with COVID-19.

A total of 331 patients with COVID-19 were included from the Third Affiliated Hospital of Sun Yat-sen University (n=18) and Huangshi Hospital of Traditional Chinese Medicine (Infectious Disease Hospital) (n=313). All patients were followed-up and then divided into death (n=23) and survival (n=308) groups (**Figure 1**). The median age of the Death and Survival groups was significantly different (69 years and 51 years, respectively, P<0.01). Analysis of clinical characteristics showed that these patients presented with fever (n=271, 81.9%), diarrhea (n=20, 6.0%) and had comorbidities (n=89, 26.9.0%). Moreover, the number of patients with older age and comorbidities in the Death group was significantly greater than in the Survival group (P<0.05) (**Table 1**). There were no significant differences in sex, clinical symptoms such as fever and diarrhea and laboratory markers WBC, HGB, and monocyte between both groups. However, the neutrophil count, NLR, INR, CRP, DBIL, UREA, LDH and GLU were significantly higher in the Death group than in the Survival group (P<0.01), while RBC, PLT, lymphocyte count and ALB levels were significantly lower in the Death group than in the Survival group (P<0.01) (**Table 1**).

**Table 1:** Baseline demographic and clinical characteristics of COVID-19 patients in Survival and Death groups.

Variable	Survival (n=308)	Death (n=23)	*p*-value
Age (years)	51.00 (39.00, 62.00)	69.00 (60.00, 84.00)	<0.01
Sex			0.83
Female	158 (51.3%)	11 (47.8%)	
Male	150 (48.7%)	12 (52.2%)	
Fever			1.00
No	56 (18.2%)	4 (17.4%)	
Yes	252 (81.8%)	19 (82.6%)	
Diarrhea			0.15
No	291 (94.5%)	20 (87.0%)	
Yes	17 (5.5%)	3 (13.0%)	
Comorbidities			0.03
No	230 (74.7%)	12 (52.2%)	
Yes	78 (25.3%)	11 (47.8%)	
WBC (×10^9^/L)	4.51 (3.58, 5.54)	5.55 (3.75, 7.35)	0.05
RBC (×10^12^/L)	4.48 (0.61)	4.08 (0.81)	<0.01
HGB (g/L)	132.04 (16.80)	125.17 (17.97)	0.06
PLT (×10^9^/L)	159.00 (130.00, 197.50)	123.00 (90.00, 176.00)	<0.01
Neutrophils (×10^9^/L)	2.82 (2.06, 3.71)	4.27 (2.71, 6.41)	<0.01
Lymphocyte (×10^9^/L)	1.13 (0.81, 1.48)	0.66 (0.46, 0.77)	<0.01
NLR	2.27 (1.54, 3.56)	7.22 (3.86, 15.93)	<0.01
Monocyte (×10^9^/L)	0.45 (0.31, 0.58)	0.36 (0.21, 0.53)	0.08
INR	0.91 (0.87, 0.95) (n=279)	0.96 (0.90, 1.04) (n=22)	<0.01
ALB (g/L)	40.60 (36.80, 43.40) (n=303)	35.60 (30.00, 38.40)	<0.01
CRP (mg/L)	20.18 (6.71, 47.74) (n=300)	53.05 (37.05, 91.13)	<0.01
DBIL (umol/L)	6.30 (4.80, 7.70) (n=303)	8.90 (6.50, 12.00)	<0.01
UREA (mmol/L)	3.71 (3.00, 4.71)	5.49 (4.50, 9.67)	<0.01
LDH (U/L)	241.00 (195.00, 312.00) (n=294)	391.00 (316.00, 574.00) (n=22)	<0.01
GLU (mmol/L)	5.71 (5.21, 6.45)	6.94 (5.80, 9.93)	<0.01

All variables with missing values are labeled with a specific number of samples.

WBC, white blood cell; RBC, red blood cell; HGB, hemoglobin; PLT, blood platelet; NLR, neutrophil-to-lymphocyte ratio; INR, international normalized ratio; ALB, albumin; CRP, C-reactive protein; DBIL, direct bilirubin; LDH, lactate dehydrogenase; GLU, glucose.

### Multivariate Cox regression analysis for 80-day survival of patients with COVID-19

**Figure 2:**
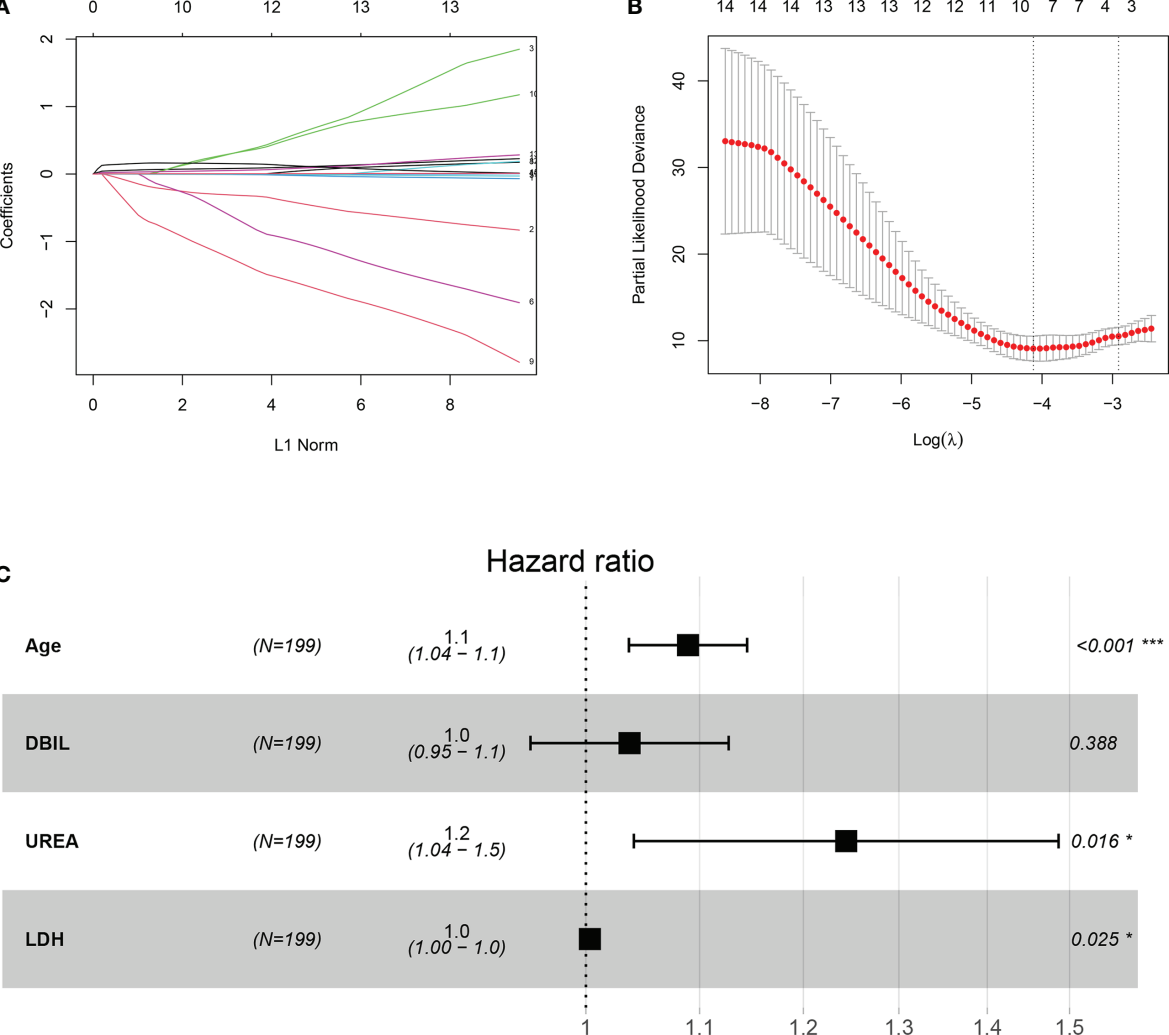
Construction of the integrated prognostic features of patients with COVID-19. **(A)** LASSO coefficient profiles of the 21 prognostic features. **(B)** Selection of the tuning parameter (lambda) in the LASSO model by fivefold cross-validation based on minimum criteria for prognosis; the lower X-axis shows log (lambda), and the upper X-axis shows the average number of prognostic features. The Y-axis indicates the partial likelihood deviance error. Red dots represent average partial likelihood deviances for every model with a given lambda, and vertical bars indicate the upper and lower values of the partial likelihood deviance errors. The vertical gray dotted lines define the optimal values of lambda, which provides the best fit. **(C)** Forest plots of multivariate Cox regression analysis for 80-day survival of patients with COVID-19. DBIL, direct bilirubin; LDH, lactate dehydrogenase.

All patients were randomly divided into training (n=199) and validation (n=132) groups. There were no significant differences in age and sex between the training and validation groups (**Supplementary Table 1**). A total of 21 features were collected from each patient. After removing irrelevant and redundant features, 14 features were retained for LASSO regression analysis (**Figures 2A, B**). The LASSO regression analysis showed that age, DBIL, UREA and LDH were predictive prognostic factors for the 80-day survival of COVID-19 patients in the training group. All these 4 features were incorporated in the multivariate Cox regression analysis. The multivariate Cox regression analysis demonstrated that age, UREA and LDH were independent prognostic factors for the 80-day survival of patients with COVID-19 (**Figure 2C**).

### Prognostic nomogram for 80-day survival of patients with COVID-19

**Figure 3:**
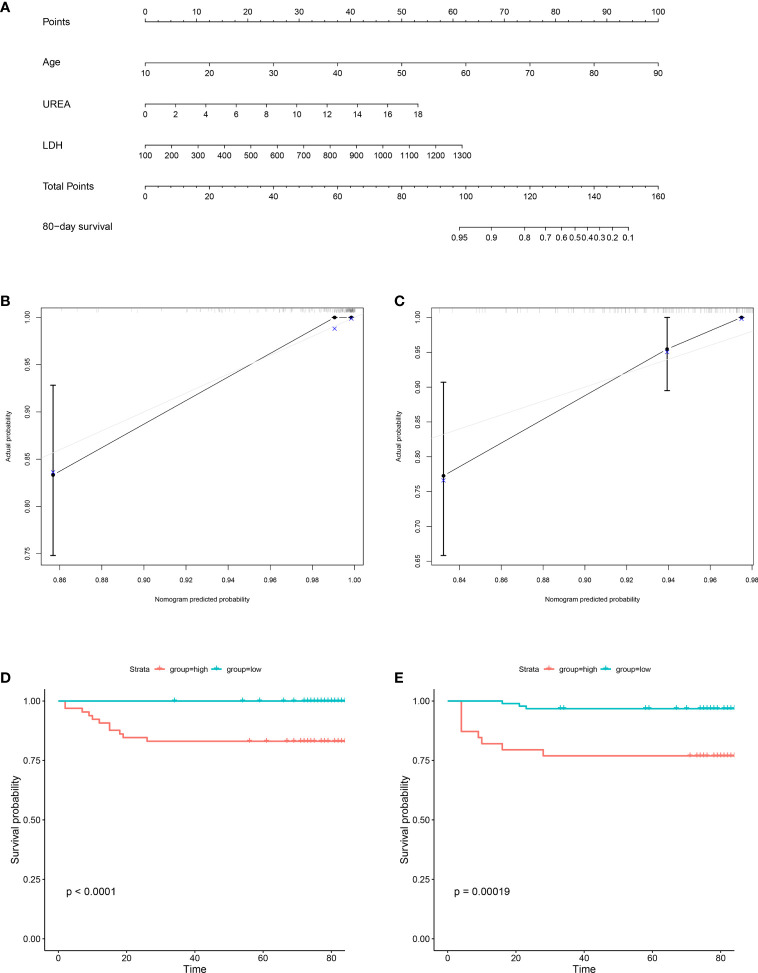
Construction of prognostic nomogram for 80-day survival of patients with COVID-19. The prognostic nomogram composed of age, UREA and LDH was developed. **(A)** Nomogram predicting 80-day survival probability in patients with COVID-19 was plotted. To use this nomogram during clinical practice, each variable axis has a separate parameter, and a line is drawn up to calculate the number of points corresponding to each parameter. The sum of these scores is located on the total point axis, and a line is drawn down along the line to get the 80-day survival probability of patients with COVID-19. **(B, C)** Calibration plot of the prognostic nomogram. The nomogram was calibrated for the probability of 80-day survival in patients with COVID-19 in the training group **(B)** and validation group **(C)**. The predicted probability of 80-day survival is plotted on the x-axis; the actual probability of 80-day survival is plotted on the y-axis (bootstrap 1,000 repetitions). **(D, E)** Kaplan-Meier Analysis for the patients with COVID-19 in the training group **(D)** and validation group **(E)**. Blue line: total points < 86.72142 (low group); red line: total points ≥86.72142 (high group).

**Figure 4:**
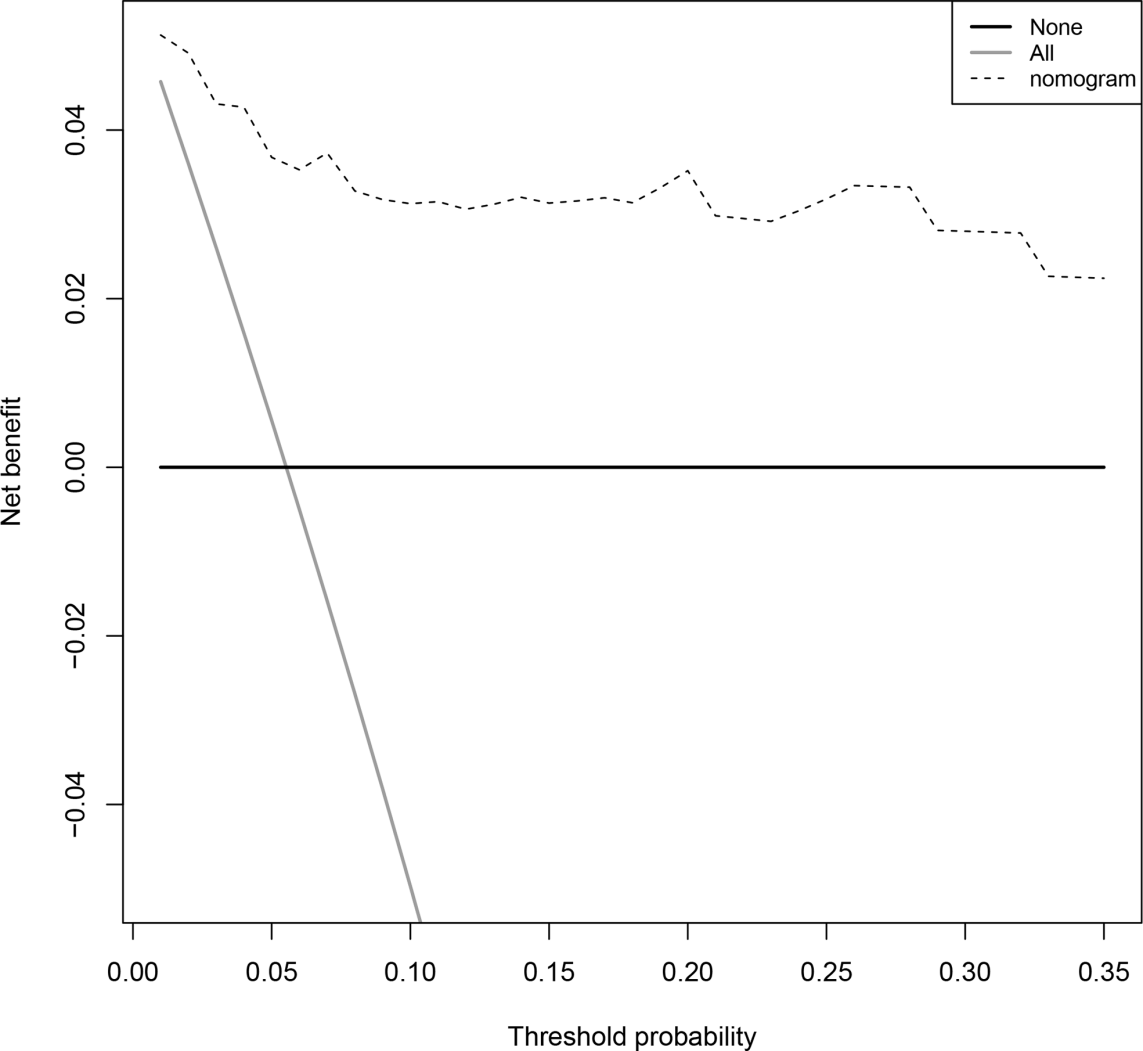
Decision curves for predicting the net benefit of the prognostic nomogram. A perfect prediction model (grey line), screen none (horizontal solid black line), and screen based on the nomogram (black dotted line).

To predict 80-day survival of patients with COVID-19, a prognostic nomogram was constructed based on the above-mentioned statistically significant independent prognostic factors (**Figure 3A**). The bootstrap-corrected concordance index (C-index) was 0.933 (0.879-0.987) and 0.894 (0.819-0.969) for the training and validation groups, respectively. The calibration plot for the probability of 80-day survival showed optimal agreement between the prediction and actual outcomes in training (**Figure 3B**) and validation (**Figure 3C**) groups. Furthermore, a total score was obtained by the sum of scores of the associated predictors and referred to as the probability of 80-day survival in the bottom axis. We divided these patients in the training group into high and low-score groups according to the optimal cut-off score value (86.72142), determined by the R package survminer. COVID-19 patients in the high-score group had a poor prognosis compared to the low-score group (**Figure 3D**). Similar results were observed in the validation group (**Figure 3E**). To evaluate the clinical applicability of our prognostic nomogram, we conducted a decision curve analysis (DCA). DCA substantiated the net clinical benefit of the prognostic nomogram in the training group (**Figure 4**).

Moreover, we compared the performance of our nomogram in predicting the survival probability of COVID-19 with other models reported in the literature (Cai’s model: age, D-dimer, CRP; Cheng’s model: UREA, age, D-dimer) (Cheng et al., 2020a; Cai et al., 2021). The parameters age, D-dimer, CRP, UREA, and LDH were available for 170 patients in the training group. Although there was no significant difference during calibration curve analysis between the 3 models (**Supplementary Figure 1**), the C-index of our model was relatively higher (0.930, 95%CI: 0.875-0.985) than that of Cai’s (0.882, 95%CI: 0.799-0.965) and Cheng’s nomogram (0.888, 95%CI:.805-0.971).

## Discussion

In this study, age, UREA and LDH were identified as independent prognostic factors for COVID-19 patients and used to construct a quantitative nomogram to predict 80-day mortality, which yielded a high C-index in the training and validation groups. The nomogram yielded a high optimal agreement between predicted and actual outcomes for predicting the risk of death by calibration curve analysis and a high clinical net benefit by decision curve analysis. We also found that our model yielded a higher C-index than previously established models reported in the literature.

An increasing body of evidence suggests that risk factors for COVID-19-related mortality include older age, higher severity of illness scores, higher C-reactive protein level, lower lymphocyte counts, secondary infection and comorbidities such as diabetes (Chen et al., 2020; Wang et al., 2020a; Zhang et al., 2020). In a previous study, we found that high LDH and UREA were associated with the risk of severe COVID-19 (Gong et al., 2020). Interestingly, in the present study, we found that both markers were also associated with mortality. The serum LDH and UREA levels were significantly higher in the Death group than in the Survival group; after adjusting for DBIL, they remained independent prognostic risk factors for COVID-19. SARS-CoV-2 infection can induce an inflammatory response and subsequent kidney damage, leading to elevated LDH and UREA. LDH is a biomarker for tissue damage and systemic inflammatory response (Kishaba et al., 2014; Ferrari et al., 2020; Kermali et al., 2020) that is often significantly elevated in COVID-19 patients and is an independent risk factor for COVID-19-associated mortality. UREA is a biomarker for kidney damage (Cheng et al., 2020b) that has also been reported as an independent risk factor for in-hospital death of COVID-19 patients. The above findings were in line with the results of the present study.

Cheng et al. established a nomogram consisting of BUN, D-dimer and age to predict the survival probability of COVID-19. Moreover, Li Cai et al.’s model included age, D-dimer and CRP. Interestingly, D-dimer was found to be an important prognostic factor and included in both models (Cheng et al., 2020a; Cai et al., 2021). In our research, D-dimer had more than a 5% missing rate and was hence excluded from further analysis. The parameters age, D-dimer, CRP, UREA, and LDH were available for 170 patients in the training group. Importantly, the C-index of our model was higher than that of Cheng’s and Cai’s, suggesting its robust predictive ability for mortality. Although a high CRP was observed in the death group compared with the survival group, LDH was selected by LASSO regression as an important feature instead of CRP. It is widely thought that the LDH concentration is a useful marker in evaluating the prognosis of different types of pneumonia, such as pneumocystis jiroveci pneumonia and community-acquired pneumonia, and a significant correlation between LDH and CRP in COVID-19 has been documented (Ashraf et al., 2022). We also observed a moderate correlation between LDH and CRP (r=0.562), which might explain why the lasso model did not recognize CRP as an important feature.

Strengths of our nomogram include its accuracy, objectivity, and simplicity. Although a high Sequential Organ Failure Assessment (SOFA) score helps identify COVID-19 patients with poor prognosis at an early stage (Moreno et al., 1999; Zhou et al., 2020), it comprises 7 factors, and one of these is a non-objective indicator—the Glasgow Coma Scale Score (GCS) (Li et al., 2019). The predictors in our proposed nomogram are relatively inexpensive and easy to obtain in clinical practice, which makes the nomogram highly practical and easily implementable. Indeed, it is highly likely that clinicians working in community hospitals can easily judge a COVID-19-infected patient’s condition by using our nomogram. Moreover, our nomogram yielded good performance in predicting survival, and its predictive accuracy (C-index) exceeded 0.9 (95%CI, 0.879-0.987) during COX regression analysis.

Importantly, we developed a practical quantitative prediction tool consisting of 3 commonly used, relatively cheap, easy-to-obtain indicators. The median follow-up time was 80 days. It was worth noting that all COVID-19 patients in the Death group experienced death within 30 days, suggesting that most people who get COVID-19 recover within a few weeks, and the same results would be obtained for predicting the 30-day mortality. In addition, our nomogram exhibited a net clinical benefit highlighting that it has huge prospects for application in clinical practice. There were some limitations in the study. First, given its retrospective nature, only 331 COVID-19 patients from two centers were included. Moreover, our nomogram was not externally validated. Therefore, more studies with larger patient cohorts are warranted to further validate our findings.

## Conclusion

This study developed an accurate prognostic nomogram to early predict mortality of COVID-19 patients. This new nomogram could help clinicians in the early screening of patients with poor prognoses and optimizing the use of available medical resources to decrease the burden on healthcare facilities.

## Data availability statement

The raw data supporting the conclusions of this article will be made available from the corresponding author by request, without undue reservation.

## Ethics statement

The studies involving human participants were reviewed and approved by Ethics Committee of Third Affiliated Hospital of Sun Yat-sen University and the Ethics Commission of Huangshi Hospital of Traditional Chinese Medicine (Infectious Disease Hospital). Written informed consent from the participants’ legal guardian/next of kin was not required to participate in this study in accordance with the national legislation and the institutional requirements.

## Author contributions

BH and JX conceived and designed this study. GH, YJ, JC, YW and SH collected the data. YC and JG drafted and critically revised the manuscript. All authors have read and approved the final manuscript. All authors contributed to the article and approved the submitted version.

## Funding

This work was supported by grants from the Science and Technology Program of Guangzhou, China [201903010039]; Guangdong Key R&D Plan of China [2020B1111160003]; Guangdong Key R&D Plan of China [2019B020231001].

## Conflict of interest

The authors declare that the research was conducted in the absence of any commercial or financial relationships that could be construed as a potential conflict of interest.

## Publisher’s note

All claims expressed in this article are solely those of the authors and do not necessarily represent those of their affiliated organizations, or those of the publisher, the editors and the reviewers. Any product that may be evaluated in this article, or claim that may be made by its manufacturer, is not guaranteed or endorsed by the publisher.
