# Supplementary material for: An early novel prognostic model for predicting 80-day survival of patients with COVID-19
Source: Front Cell Infect Microbiol. 2022 Oct 27;12:1010683. doi: 10.3389/fcimb.2022.1010683 (PMC9647191; doi:10.3389/fcimb.2022.1010683)
Supplement: Supplementary file 1 [file DataSheet_1.docx]

**Supplementary Table 1. Baseline Characteristics of the Study Cohort**

|  | Training group | Validation group | p-value |
| --- | --- | --- | --- |
| N | 199 | 132 |  |
| Gender |  |  | 0.91 |
| Female | 101 (50.8%) | 68 (51.5%) |  |
| Male | 98 (49.2%) | 64 (48.5%) |  |
| Age(year) | 51.00 (40.00, 65.00) | 53.00 (39.00, 63.00) | 0.99 |
| Basic disease |  |  | 0.26 |
| No | 150 (75.4%) | 92 (69.7%) |  |
| Yes | 49 (24.6%) | 40 (30.3%) |  |

**Supplementary Figure 1.**


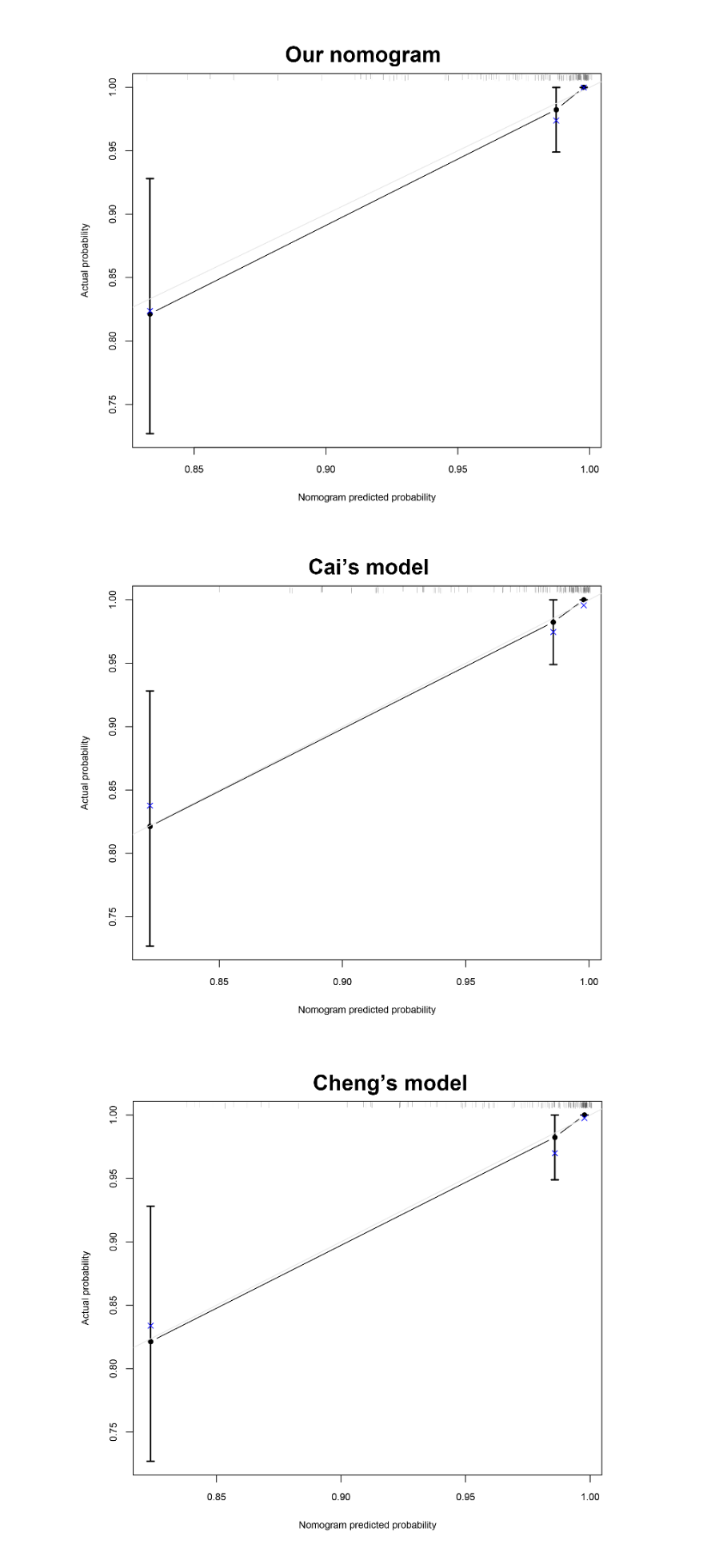


**Supplementary Figure 1.** Calibration plot of our nomogram and other’s established nomograms for the probability of 80-day survival in patients with COVID-19.
